# Modelling decay in effectiveness for evaluation of behaviour change interventions: a tutorial for public health economists

**DOI:** 10.1007/s10198-021-01417-7

**Published:** 2021-12-16

**Authors:** Paolo Candio, Koen B. Pouwels, David Meads, Andrew J. Hill, Laura Bojke, Claire Williams

**Affiliations:** 1 Centre for Economics of Obesity, Institute of Applied Health Research, University of Birmingham, Birmingham, B15 2TT UK; 2 Health Economics Research Centre, University of Oxford, Oxford, UK; 3 Leeds Institute of Health Sciences, University of Leeds, Leeds, UK; 4 Centre for Health Economics, University of York, York, UK

**Keywords:** Effect decay, Mathematical modelling, Public health, Decision-making, Structural uncertainty, C6, D04

## Abstract

**Background and purpose:**

Recent methodological reviews of evaluations of behaviour change interventions in public health have highlighted that the decay in effectiveness over time has been mostly overlooked, potentially leading to suboptimal decision-making. While, in principle, discrete-time Markov chains—the most commonly used modelling approach—can be adapted to account for decay in effectiveness, this framework inherently lends itself to strong model simplifications. The application of formal and more appropriate modelling approaches has been supported, but limited progress has been made to date. The purpose of this paper is to encourage this shift by offering a practical guide on how to model decay in effectiveness using a continuous-time Markov chain (CTMC)-based approach.

**Methods:**

A CTMC approach is demonstrated, with a contextualized tutorial being presented to facilitate learning and uptake. A worked example based on the stylized case study in physical activity promotion is illustrated with accompanying R code.

**Discussion:**

The proposed framework presents a relatively small incremental change from the current modelling practice. CTMC represents a technical solution which, in absence of relevant data, allows for formally testing the sensitivity of results to assumptions regarding the long-term sustainability of intervention effects and improving model transparency.

**Conclusions:**

The use of CTMC should be considered in evaluations where decay in effectiveness is likely to be a key factor to consider. This would enable more robust model-based evaluations of population-level programmes to promote behaviour change and reduce the uncertainty surrounding the decision to invest in these public health interventions.

**Supplementary Information:**

The online version contains supplementary material available at 10.1007/s10198-021-01417-7.

## Introduction

Behaviour change interventions play an important role in improving population health [1, 2]. Public health decision makers are increasingly interested in the evaluation of these programmes to inform resource allocation decisions [3]. In these evaluations, however, is estimating long term intervention effects (e.g., changes in physical activity (PA) habits) is often a challenge [4]. Effectiveness evidence tends to be short-term [5], whilst most health benefits accrue over the long term [6]. Acknowledging this issue, the National Institute for Health and Care Excellence (NICE) recommends the application of extrapolation methods and careful consideration in defining model assumptions that ought to be plausible and related uncertainties that need to be fully explored and systematically addressed [7].

In practice, however, modelling studies in public health have commonly used discrete-time frameworks, such as Markov chains [8]. In its basic form, Markov chains simulate how a cohort of individuals move between predefined states at fixed transition probabilities [9].

Although, in principle, discrete-time Markov chain-based models can be populated with different transition probabilities for each of the different model cycles, lack of long-term follow-up data has meant the use of poorly justified and often implicit assumptions regarding the decay in intervention effects over time. A recent review found that intervention effects were assumed to remain constant over time in most instances, often without them being subject to any decay (15 of the 25 reviewed studies), even when lifetime time horizons were considered [10].

As an example, that review found that in a 2012 economic appraisal commissioned by the Department of Health to NICE to develop guidance on environmental interventions that promote PA, intervention effects were assumed to remain at 100% over time for the entire exposed population [11]. This was acknowledged by the authors as a main limitation. Where this assumption was relaxed, for instance by assuming that intervention effects were fully sustained for the first year and then subsequently to remain constant over time at a 33% or 50% of the initial effect [12, 13] (i.e., linear distributions), the review found that, in most studies, robustness checks were not adequately performed. This is problematic because intervention effects are likely to follow non-linear, rebound trajectories [14] and the validity of study findings is likely to depend heavily on how intervention-induced changes in behaviours are sustained over time [15].

Although the ideal solution would be to collect long-term data on the maintenance of intervention-induced changes over time by different individuals, in the absence of such data, we argue that continuous-time Markov chains (CTMC) represent a theoretically superior modelling option in these modelling settings and an optimal technical solution to the issue at hand. Instead of using tunnel states to account for time-dependence, which can make a model overly complex and impractical, CTMC can formally capture time-dependent intervention effects using statistical distributions. This enables greater flexibility in the choice of still hypothetical yet more plausible rebound trajectories and assessing their implications for study findings.

Although CTMC methods have been used to study disease progression, the current lack of a practical guide may explain their limited application currently in public health policy research [16]. Therefore, with the aim of demonstrating the utility of CTMC, this paper presents a tutorial on this modelling solution placed in the context of population-level interventions to promote healthy behaviours. After introducing the reader to key concepts relating to the CTMC modelling framework, a step-by-step guide is presented on how to implement this method in practice. To contextualize learning, a worked example based on a stylized case study in PA promotion is illustrated to demonstrate the framework principles and its functionality, with the accompanying R code provided.

## Modelling framework

Let us consider a population of healthy individuals (i.e., healthy state) grouped into four ordinal sub-categories of health behaviours. Considering physical activity, (PA): inactive (1), insufficiently active (2), moderately active (3) and active (4) [17]. The healthy state can be seen as a macro state, whose composition is dependent on the frequency distribution of the inner micro-states (the four PA levels). The probability (*P*) that the population, on average, move from the healthy to a disease state is therefore equal to the weighted average of the four PA-level risks (*p*_*i*_, *i* = PA level):

$$P = \frac{{\mathop \sum \nolimits_{i = 1}^{4} (p_{i } w_{i} )}}{{\mathop \sum \nolimits_{i = 1}^{4} w_{i} }}\quad \quad \sum\nolimits_{(i = 1)}^{4} {w_{i} } \;{\text{always}}\;{\text{adding}}\;{\text{up}}\;{\text{to}}\;{1}{\text{.}}$$

*w*_*i*_ proportion of individuals in a PA state *i*, relative to the total population, at any point in time.

## Transition between physical activity states

The healthy state can be represented as an embedded Markov chain (EMC), also known as nested MC or embedded jump chain [9]. EMC methods, an extension of discrete-time MCs, have been applied in many fields to capture complex system-level behaviours [16]. Given an EMC structure, any transition between the four PA levels (i.e., 16 possible transitions) and two time points, *t* =  − 1 (baseline) and *t* = 0 (6 months post-intervention), can be described, using a matrix algebra framework, by a square matrix of transition probabilities.

The values on the diagonal of the matrix represent the four possible transitions from and to the *same state* (*P*_r_), while the off-diagonal cells include the transitions between a given state and any other PA states (*P*_m_). No change in PA level between *t* − 1 and *t* = 0 is therefore a four-by-four identity matrix, where all the diagonal values are equal to one, and the remaining values are all zeros.

**Fig. 1:**
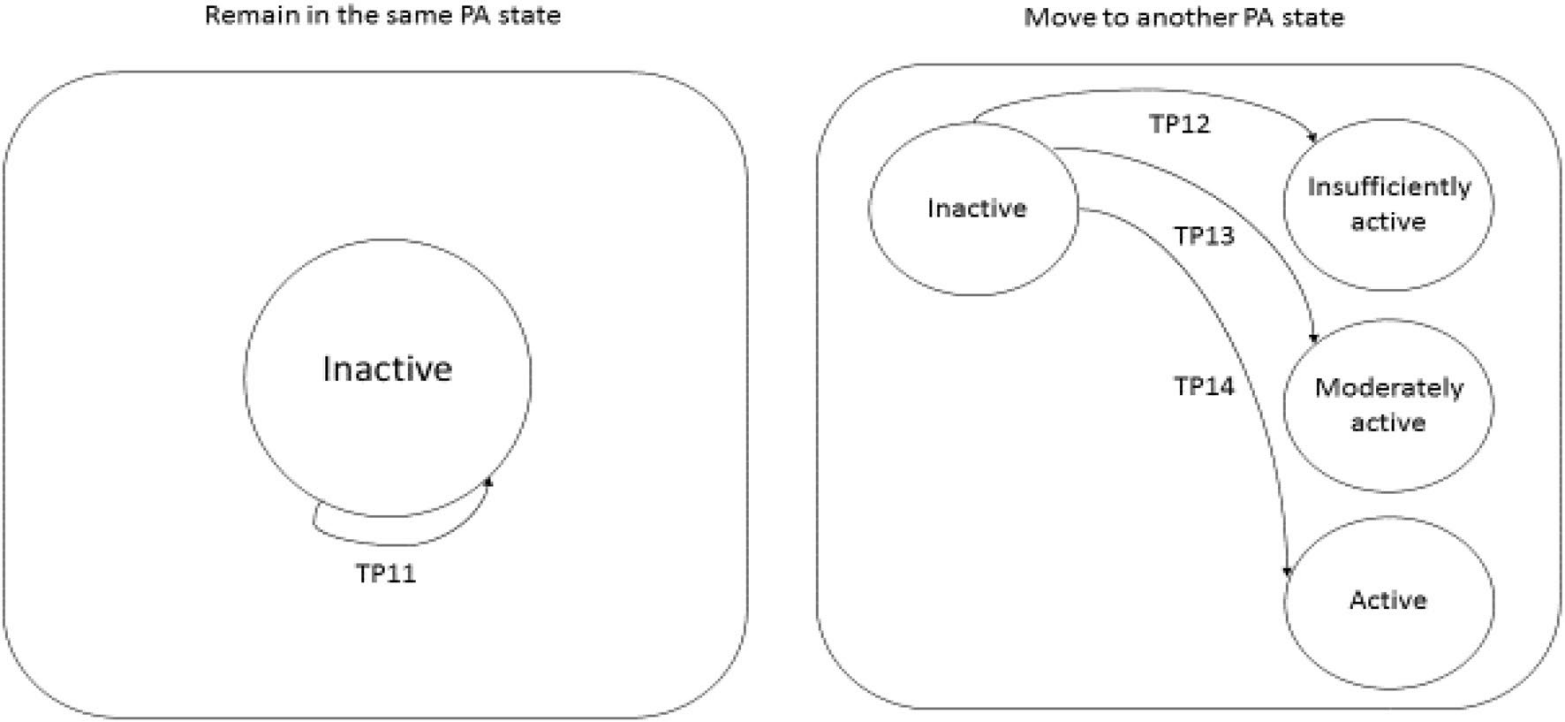
Possible transitions from the inactive state

For each of the four PA states, there is only one possible transition from and to the same state (*P*_r_ = TP_11_) and three possible transitions to the three other levels (*P*_m_ = TP_12_ + TP_13_ + TP_14_, Fig. 1). *P*_r_ and *P*_m_ are two complementary events, with a combined probability that must always be equal to 1.

## Population-level impact

An intervention-induced change in behaviour represents a shift in the transition probabilities from their “natural” course (*P*_nat_^−1,0^). In matrix terms, given a matrix *A*_*t*−1,*t*_, namely, the *intervention effect matrix* for the period *t* =  − 1 to *t* = 0, four PA levels [1–4], and *P*_*i,j*_ = probability that the chain will move to state *j* given that is in now in state *i*, then:

$$A_{ - 1,0} = \left[ {\begin{array}{*{20}c} { - a_{1} } & {a_{1} P_{1,2} } & {a_{1} P_{1,3} } & {a_{1} P_{1,4} } \\ {a_{2} P_{2,1} } & { - a_{2} } & {a_{2} P_{2,3} } & {a_{2} P_{2,4} } \\ {a_{3} P_{3,1} } & {a_{3} P_{3,2} } & { - a_{3} } & {a_{3} P_{3,4} } \\ {a_{4} P_{4,1} } & {a_{4} P_{4,2} } & {a_{4} P_{4,3} } & { - a_{4} } \\ \end{array} } \right]$$

where *a*_*i*_* differential* transition probability (from *P*_nat_^−1, 0^), which is expressed as a rate of transition out of state *P*_*i*_. The term *a*_*i*_* P*_*i,j*_ can be interpreted as the differential rate of transition between different PA states, under the condition that *a*_*i*_* P*_m_ =  − *a*_*i*_*P*_r_.

If *a*_*i*_ is constant over time, a discrete-time chain is represented. In regression terms, *a*_*i*_ represents the intervention effect estimated from an ordered logit model. Given a baseline distribution of PA states represented by the vector θ^−1^, to obtain the post-intervention distribution of PA states θ^0^, a simple matrix multiplication is needed:

1 $$\theta^{0} = \theta^{ - 1} \times P_{ - 1,0}^{{{\text{nat}}}} \times A_{ - 1,0}$$

## Behaviour change maintenance

Following an initial change in behaviour, most individuals in the population will likely converge to their natural course of PA at different rates [14]. In other words, any effect on the behaviour will not likely remain constant over time (i.e., *A*_−1,0_ is not an identity matrix), instead it will rebound, heterogeneously depending on individual characteristics. These rebound trajectories can be partitioned into sub-segments corresponding to discrete time periods (i.e., Markov cycles). The principle is to view maintenance of behaviour change as a survival function, whereby survivorship is the *residual intervention effect* up to a certain point in time. Using the notation above, if:

*A*_−1,0_ = intervention effect matrix (100% of the effect at time t), then:

*P*_t,_^res^ = residual intervention matrix at cycle *u.*

**Fig. 2:**
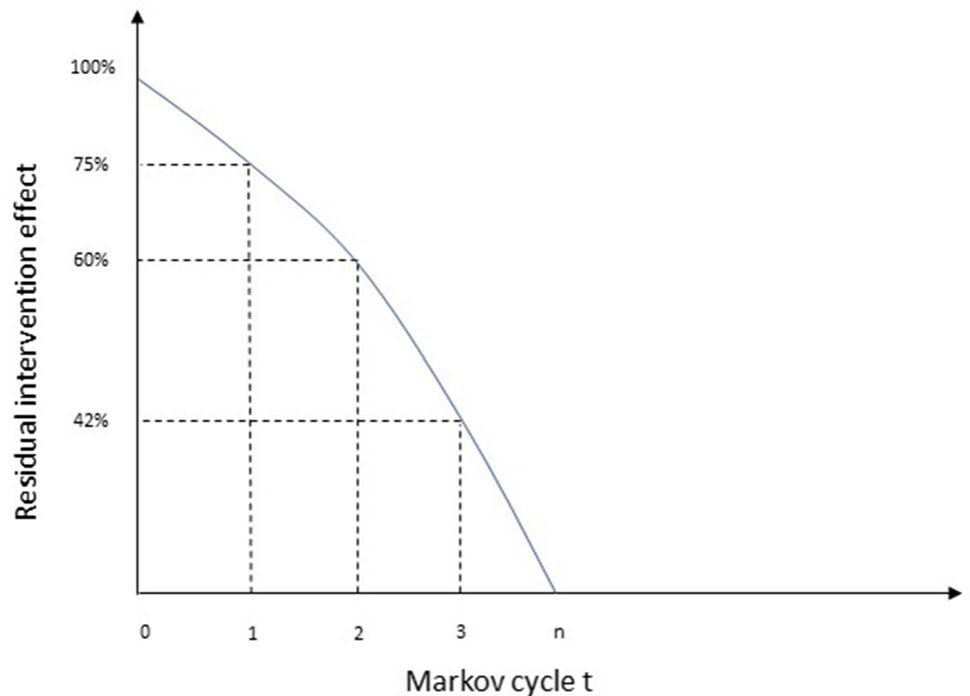
Example of maintenance of behaviour change over time (rebound trajectory)

A hypothetical representation of exponential rebound effect is given in Fig. 2.

The residual intervention effect is on the *y* axis, and the number of Markov cycles elapsed on the *x* axis. From time *t* = 0 (post-intervention, 100% of the effect), the intervention effect starts converging gradually towards zero. In this example, at the beginning of the first cycle, 25% of the intervention effect is decayed. In other words, the residual intervention effect is at 75% of its original magnitude (*P*_0,1_^res^ = 0.75). In cycle 2, 40% of the initial effect has faded out (*P*_0,2_^res^ = 0.60), corresponding to a 20% loss of intervention effect from the previous cycle (15/75), and so on.

To compute the residual intervention effect (*P*_0,*u*_^res^) at any given point in time (cycle), three steps need to be followed. First, a parametric survival model needs to be estimated. The estimated rates of decay (λ) are computed *for each of the four PA levels* (i) and *for the cycles elapsed up to cycle u*. Taking an example of decay of effect between two time points, these rates can be converted into probabilities using the following formula [15]:

2 $$p\lambda_{i} \left( {u - 1, \, u} \right) = 1 - \exp \left[ {H\left( {u - 1} \right) - H\left( u \right)} \right]$$

*H*(*u*) = cumulative hazard at cycle *u*. For example, if a Weibull distribution is assumed for the hazard, $H\left( u \right) = \lambda_{i} \left( u \right)^{\gamma } :p\lambda_{i} \left( {u - 1, \, u} \right) = 1 - \exp \left[ {\lambda_{i} \left( {u - 1} \right)^{\gamma } - \lambda_{i} \left( u \right)^{\gamma } } \right].$ Probability formulae for other standard distributions are shown in Appendix I.

To calculate the probability of residual effect from the previous cycle for a given PA state *i*:

3 $$p_{i}^{{{\text{res}}}} \left( {u - 1,u} \right) = 1 \, - \, p\lambda_{i} \left( {u - 1,u} \right)$$

Hence, the general formulation for calculating the probability of residual intervention effect left up to cycle *u* from time *t* = 0:

4 $$P_{0,u}^{{{\text{res}}}} = \mathop \prod \limits_{0}^{u} \left( {p_{i}^{{{\text{res}}}} \left( {t,u} \right)} \right)$$

In other words, a matrix multiplication of the cycle probabilities of residual intervention effect from time *t* up to cycle *u* is needed. Using the example in Fig. 1, once $P_{0,u}^{{{\text{res}}}}$ is computed, for example, for *u* = 3 (i.e., $P_{0,u}^{{{\text{res}}}} = 0.42$), the residual intervention effect matrix for cycle 3 is obtained by multiplying the intervention effect matrix (*A*_−1,0_) by *each* of the respective cycle probabilities of residual effect (*p*_*i*_^res^) up to cycle 3. Thus, a series of subsequent PA transition probability matrices (from cycle *t* = 0 to cycle *u*) incorporating the progressive loss of intervention effect over time can be calculated. In notation terms:

5 $$\theta^{u} = \theta^{u - 1} *P\left( {_{0,u - 1}\,^{{{\text{res}}}} } \right)^{ - 1} *P\left( {_{0,u}\,^{{{\text{res}}}} } \right)$$

where $\theta^{u - 1} *P\left( {_{0,u - 1}\,^{{{\text{res}}}} } \right)^{ - 1} = \theta^{0} ,$ that is the post-intervention distribution of PA states (i.e., 100% of intervention effect). Using the example in Fig. 2, the PA distribution for the first three cycles is calculated as follows:

- For cycle 1:
  $$\theta^{{1}} = \theta^{0} *A_{{ - {1},0}}^{ - 1 } * P\left( {_{{0,{1}}}\,^{{{\text{res}}}} } \right)$$
- For cycle 2:
  $$\theta^{2} = \theta^{1 } *P\left( {_{0,1}\,^{{{\text{res}}}} } \right)^{ - 1} *P\left( {_{0,2}\,^{{{\text{res}}}} } \right)$$
- For cycle 3:
  $$\theta^{3} = \theta^{2 } * \, P\left( {_{0,2}\,^{{{\text{res}}}} } \right)^{ - 1} *P\left( {_{0,3}\,^{{{\text{res}}}} } \right)$$

The steps illustrated above show that to calculate the PA distribution at any given cycle, the residual intervention effect from the previous cycle is first subtracted (through matrix inversion), and then replaced by the current cycle’s residual intervention effect (through matrix multiplication). These calculations can be repeated for any given population sub-group the evaluation ought to consider (e.g., by socio-economic status) and then combined based on, for instance, group size (i.e., weighted average).The number of chosen subgroups, however, will inevitably increase the computational task.

## Illustration of the modelling framework

A stylised case study of a population-level physical activity intervention designed based on the previous implementations is used as an exemplar [15, 18]. Let’s All Get Fit (LAGT) is a city-wide programme which offers free access to gym sessions and community sport events to encourage residents, especially those sedentary and from low socio-economic backgrounds, to become more active. The aim is to estimate the health benefits deriving from changes in the distribution over time of the four PA levels generated, in two population sub-groups (i.e., high and low income).

For the ease of illustration, aligning with Fig. 2, the extrapolated time horizon is 30 months. Only baseline (*t* =  − 1) and 6-month post-intervention (*t* = 0) data on PA levels are available. Hence, extrapolation of the intervention effect is needed over four cycles of six months each, post the observed follow-up at 6 months. A Weibull distribution, which was previously fitted to gym attendance data from the same study [15], was conveniently used as a proxy measure of the decay in intervention effectiveness over the remaining 24 months (i.e., rebound trajectory). The population (*N* = 6000 adults) consists of eight sub-groups, that is, two levels of socio-economic status (high income = 4000 and low income = 2000) each divided into four PA levels. At baseline (*t* =  − 1), the frequency distribution of PA (θ^−1^, see Eq. 1) by socio-economic status is shown in Table 1.

**Table 1:** Baseline distribution of physical activity levels

*N* = 6000	Inactive	Insufficiently active	Moderately active	Active
High-income group *n* = 4000	15% (600)	20% (800)	30% (1200)	35% (1400)
Low-income group n = 2000	25% (500)	30% (600)	25% (500)	20% (400)

## Initial change in behaviour

The intervention effect matrices are thus estimated (for the first 6 months) fitting an ordered logistic regression model, (*A*_*t*−1,*t*_, see Eq. 1) for each of the two socio-economic sub-groups (Tables 2 and 3).

**Table 2:** High-income subgroup intervention effect matrix

High-income group	Inactive	Insufficiently active	Moderately active	Active
Inactive	15%	48%	31%	6%
Insufficiently active	8%	39%	42%	11%
Moderately active	3%	22%	51%	24%
Active	2%	11%	44%	43%

**Table 3:** Low-income subgroup intervention effect matrix

Low-income group	Inactive	Insufficiently active	Moderately active	Active
Inactive	45%	38%	16%	1%
Insufficiently active	9%	40%	41%	10%
Moderately active	2%	14%	47%	37%
Active	3%	20%	50%	27%

The frequency distribution of PA levels at the first follow-up (θ^0^) is thus simply obtained via matrix multiplication of θ^−1^ by the respective *A*_−1,0_, with the following results:

In this hypothetical example (Table 4), the intervention induced heterogeneous changes in PA levels between sub-groups. The proportion of inactive adults increased similarly across the two groups, with 9.55% and 9.95% fewer inactive adults post intervention for the high-income and the low-income subgroups, respectively. Conversely, the proportion of active adults remained stable within the low-income subgroup (from 20% to 17.90%), whereas it saw a 9.65% decrease in the high-income subgroup.

**Table 4:** Distribution of physical activity at follow-up (6 months)

*N* = 6000	Inactive	Insufficiently active	Moderately active	Active
High-income group *n* = 4000	5.45% (218)	25.45% (1.018)	43.75% (1.750)	25.35% (1.014)
Low-income group n = 2000	15.05% (301)	29.00% (580)	38.05% (761)	17.90% (358)

## Behaviour change maintenance

The post-intervention distribution of PA levels in Table 4 incorporates the change in behaviour induced by the LAGT (Tables 2 and 3) in absence of which Table 4 would have matched the values showed in Table 1 (i.e., “natural” levels of PA assumed to be constant over time, *P*_nat_^−1 0^ is an identity matrix).

If the change in behaviour induced by the intervention remained constant over time, then the values shown in Table 4 would not vary in the subsequent time periods. Conversely, if the residual effect of LAGT were equal to zero following the initial change, then the distribution of PA levels would be equal to baseline. However, as hypothesised above, the initial change in behaviour is likely to gradually converge to the natural course for most individuals in the population over time.

As a first step, following Eq. (2) and based on the Weibull distribution fitted to the gym attendance data mentioned above (proxy measure of behaviour change maintenance over time), a series of probabilities of effect decay between *t* and *u*, for each subgroup and cycle elapsed are calculated (by converting the respective rates λ estimated from analysis of the attendance data). To obtain the residual effect probabilities, each of these values are subtracted from 1 (see Eq. 3). Taking the group of high income inactive as an example, this probability was equal to 0.017. In other words, of the initial change in behaviour observed between *t* =  − 1 and *t* = 0, 98.3% of the effect is still left at the *t* = 1 (Appendix II).

Now that we have calculated the residual effect probabilities, to obtain the *t* = 1 distribution of PA levels, we need to follow Eq. (5). That is, for the high-income inactive group, subtracting 1.7% of the effect from the PA distribution at time *t* = 0. This is obtained by multiplying the PA distribution shown in Table 4 by the inverted intervention effect matrix [(*A*_−1,0_)^−1^] and the residual probabilities matrix. Figure A (Appendix III). illustrates the passages described above for the high-income inactive group.

For the subsequent cycle *t* = 2, the transition probabilities belonging to *P*_m_ need each to be multiplied by the probability of effect decay between *t* = 1 and *t* = 2. In the LAGT study, this was equal to 0.057 for the high-income inactive group (Appendix II). Figure B (Appendix III) shows the steps for how to calculate the PA distribution for the high-income inactive group at time = 2, that is two cycles after the intervention ended. This process is repeated until the residual effect probability matrix is either assumed or estimated to be an identity matrix (i.e., no residual effect of the intervention, Markov cycle *n* in Fig. 2).

## Discussion

This paper is concerned with the issue of modelling the decay in effectiveness over time of population-level behaviour change interventions. To operationalise this, we present a CTMC-based practical tutorial which, through a step-by-step approach, illustrates the mathematical structure of the framework and how it can be implemented.

We believe that the proposed framework presents a simple and flexible modelling solution to address some of the limitations of discrete-time Markov chains. It provides the analyst with greater flexibility of choice of statistical distributions reflecting more plausible—yet unknown—long-term effectiveness trajectories for different interventions and individuals. Indeed, it can be readily adapted for capturing heterogeneous intervention effect trajectories by different sub-groups (e.g., socio-economic status) and be consequently used to inform assessments regarding the health equity impact of population-level interventions [19]. Furthermore, by formally requiring a choice of distribution, and therefore assumption regarding the decay in effectiveness over time, the use of CTMC will improve reporting of these evaluation studies and facilitate peer-review processes.

Unlike more advanced modelling techniques (e.g., discrete event simulation), the proposed approach presents a relatively small incremental change from current practice and does not require high-level modelling or programming skills. In addition, it lends itself to ready implementation and adaptation to different decision problems, with potential for a widespread used in public health settings. To this purpose, we have provided a worked example in R programming (Appendix IV).

However, the proposed framework is not without limitations. Being based on the Markov paradigm, probabilities of transition to future states are dependent only on the present state (i.e., memory-less property) [9]. In the context of behaviours, especially when evaluating short time trajectories and sensitive life phases (e.g., retirement), this is a limitation as future events are unlikely to be independent from previous experience [20]. A possible solution to this is the application of tunnel states, which can enable the integration of experience from previous cycles [21]. However, the choice to incorporate tunnel states will depend on the decision problem, as well as the ability to balance complexity and practicality, as the model can become difficult to manage, especially if implemented in a spreadsheet [22].

The proposed approach assumes that the health states represent homogeneous groups of individuals. To this respect, an individual level framework, such as microsimulation [23], can be more suitable, provided relevant data are available. Lack of long-term follow-up data is a common hurdle of public health evaluations, not least the information regarding the sustainability of intervention effects [5]. Nevertheless, this does not justify the use of implausible assumptions and lack of robustness checks.

The proposed extrapolation approach is merely deterministic and based on a proxy measure of behaviour change maintenance which may not be always available. Alternative sources of information on plausible rebound trajectories, either hypothetical distributions, obtained from simulated data, or based on information elicited by experts (e.g., personal trainers) can be used and tested with the CTMC framework. In addition, the illustration provided here is based on a single Weibull-shaped rebound trajectory and evaluation studies should use scenario analysis comparing different plausible rebound trajectories to fully explore and address the related uncertainty.

Furthermore, the issue of heterogeneity has been addressed only in terms of differences in intervention effect between individual characteristics over time. However, heterogeneity encompasses a much broader spectrum of issues to include, for example, differences in the way individuals benefit from behaviour change, which has not been covered here. However, we believe that this paper can help raise awareness of this issue and promote methodological guidance development toward enhancing public health evaluation practices.

## Conclusions

Formally modelling the decay of effectiveness over time is important to enable more robust model-based evaluations of population-level programmes to promote behaviour change. The proposed modelling framework presents a simple solution to overcome some of the limitations of commonly used modelling paradigms and should be considered in evaluations where decay of effectiveness of the intervention is likely to be a key factor to consider.

## Supplementary Information

Below is the link to the electronic supplementary material.

Supplementary file1 (DOCX 207 KB)

## Data Availability

No data are available.

## Code availability

Software code for implementing the proposed framework using R is provided.
