# Supplementary material for: Modelling decay in effectiveness for evaluation of behaviour change interventions: a tutorial for public health economists
Source: Eur J Health Econ. 2021 Dec 16;23(7):1151–7. doi: 10.1007/s10198-021-01417-7 (PMC9395462; doi:10.1007/s10198-021-01417-7)
Supplement: Supplementary file 1 — Supplementary file1 (DOCX 207 KB) [file 10198_2021_1417_MOESM1_ESM.docx]

# Appendix I Survival distributions*


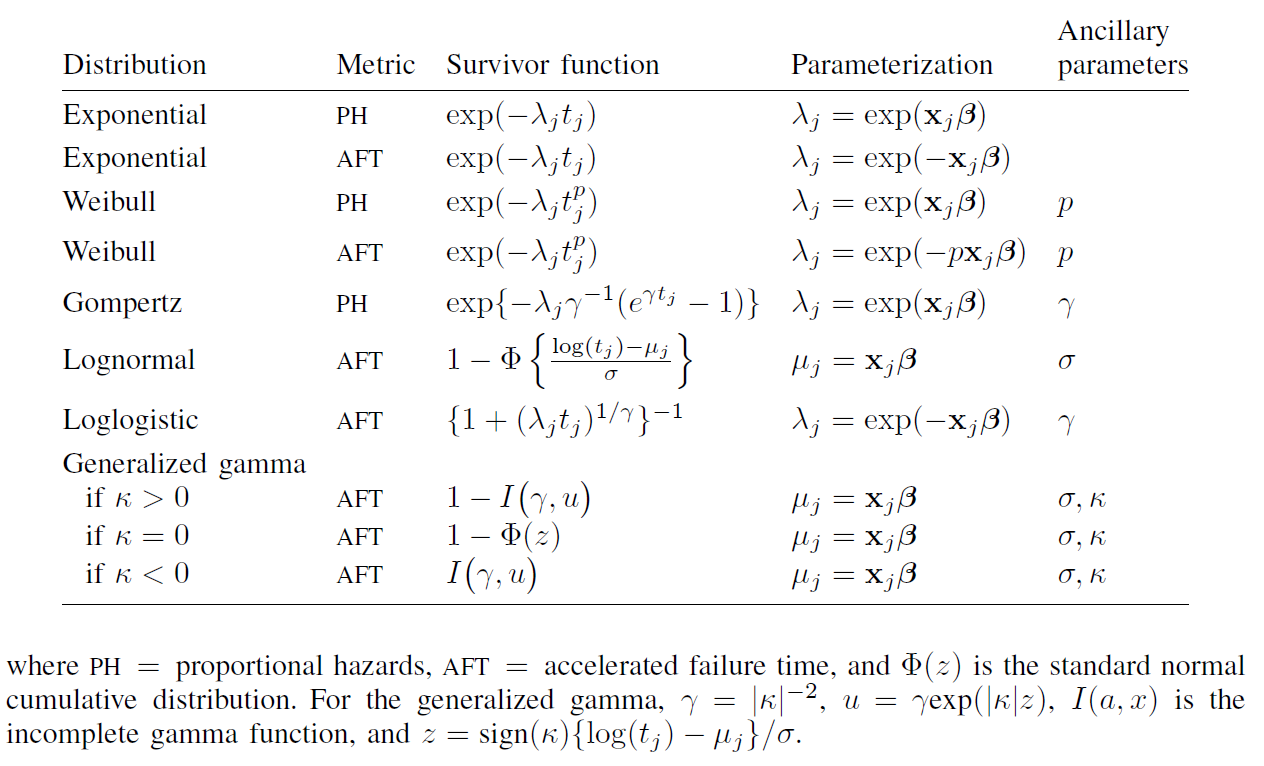


*StataCorp. 2021. Stata survival analysis. Reference Manual, Release 17. Station, TX: StataCorp LLC. [Available from: <https://www.stata.com/manuals/st.pdf>]. Access date: 31.10.2021.

# Appendix II – Decays in and residual effectiveness

**Table A** **Decays in effectiveness from time 0**

| **Markov cycle** | **inaHIGH** | **inaLOW** | **insHIGH** | | **insLOW** | **modHIGH** | **modLOW** | **actHIGH** | **actLOW** |
| --- | --- | --- | --- | --- | --- | --- | --- | --- | --- |
| 1 | 0.017 | 0.017 | | 0.015 | 0.015 | 0.013 | 0.013 | 0.010 | 0.010 |
| 2 | 0.057 | 0.060 | | 0.051 | 0.052 | 0.045 | 0.046 | 0.034 | 0.035 |
| 3 | 0.102 | 0.107 | | 0.091 | 0.093 | 0.080 | 0.082 | 0.062 | 0.063 |
| **Note**: 8 physical activity (4 categories: ina=inactive; ins=insufficiently active; mod=moderately active; act=active) / socio economic status (2 categories: HIGH=high-income; LOW=low-income) subgroups | | | | | | | | | |
|  |  |  | |  |  |  |  |  |  |
|  |  |  | |  |  |  |  |  |  |
| **Table B** **Residual effectiveness from time 0** | | | | | |  |  |  |  |
| **Markov cycle** | **inaHIGH** | **inaLOW** | | **insHIGH** | **insLOW** | **modHIGH** | **modLOW** | **actHIGH** | **actLOW** |
| 1 | 0.983 | 0.983 | | 0.985 | 0.985 | 0.987 | 0.987 | 0.990 | 0.990 |
| 2 | 0.943 | 0.940 | | 0.949 | 0.948 | 0.955 | 0.954 | 0.966 | 0.965 |
| 3 | 0.898 | 0.893 | | 0.909 | 0.907 | 0.920 | 0.918 | 0.938 | 0.937 |

**Note**: see Table A

# Appendix III – Calculations of decay in effectiveness

**Figure A** Calculations of decay in effectiveness – **time 1**: high-income group

1. Physical activity distribution of at **time 0** (post-intervention) – high-income group

| HIGH INCOME | **INA** | **INS** | **MOD** | **ACT** |
| --- | --- | --- | --- | --- |
|  | 5.45% | 25.45% | 43.75% | 25.35% |

1. Inverted intervention matrix between time -1 and time 0

| HIGH INCOME | **INA** | **INS** | **MOD** | **ACT** |
| --- | --- | --- | --- | --- |
| **INA** | 28.34 | -50.39 | 31.94 | -8.89 |
| **INS** | -8.46 | 20.68 | -16.09 | 4.87 |
| **MOD** | 3.06 | -8.81 | 11.16 | -4.40 |
| **ACT** | -2.28 | 6.07 | -8.79 | 5.99 |

By multiplying A by B, the physical activity distribution of at time -1 (baseline) is obtained:

| HIGH INCOME | **INA** | **INS** | **MOD** | **ACT** |
| --- | --- | --- | --- | --- |
|  | 15% | 20% | 30% | 35% |

1. Residual effect-adjusted transition probabilities between time -1 and time 1

| HIGH INCOME | **INA** | **INS** | **MOD** | **ACT** |
| --- | --- | --- | --- | --- |
| **INA** | 16% | 47% | 30% | 6% |
| **INS** | 8% | 40% | 41% | 11% |
| **MOD** | 3% | 22% | 52% | 24% |
| **ACT** | 2% | 11% | 44% | 44% |

**Note**: values obtained by multiplying the intervention effect transition probabilities by the residual effects showed in Appendix I, Table B, first row.

By multiplying the baseline physical activity distribution by C, we finally obtain the time=1 (one cycle after the intervention ended) physical activity distribution shown at point D:

1. Physical activity distribution of at **time 1** – high-income group1

| HIGH INCOME | **INA** | **INS** | **MOD** | **ACT** |
| --- | --- | --- | --- | --- |
|  | 5.62% | 25.39% | 43.59% | 25.41% |

**Figure B** Calculations of decay in effectiveness – **time 2**: high-income group

1. Physical activity distribution of at **time 1** (post-intervention) – high-income group

| HIGH INCOME | **INA** | **INS** | **MOD** | **ACT** |
| --- | --- | --- | --- | --- |
|  | 5.62% | 25.39% | 43.59% | 25.41% |

1. Inverted intervention matrix between time -1 and time 1

| HIGH INCOME | **INA** | **INS** | **MOD** | **ACT** |
| --- | --- | --- | --- | --- |
| **INA** | 16.78 | -26.82 | 14.56 | -3.52 |
| **INS** | -4.49 | 12.20 | -9.39 | 2.68 |
| **MOD** | 1.41 | -5.09 | 7.91 | -3.22 |
| **ACT** | -1.05 | 3.26 | -6.22 | 5.01 |

By multiplying E by F, the physical activity distribution of at time -1 (baseline) is again obtained:

| HIGH INCOME | **INA** | **INS** | **MOD** | **ACT** |
| --- | --- | --- | --- | --- |
|  | 15% | 20% | 30% | 35% |

1. Residual effect-adjusted transition probabilities between time -1 and time 2

| HIGH INCOME | **INA** | **INS** | **MOD** | **ACT** |
| --- | --- | --- | --- | --- |
| **INA** | 21% | 45% | 29% | 6% |
| **INS** | 7% | 43% | 39% | 10% |
| **MOD** | 3% | 21% | 54% | 23% |
| **ACT** | 2% | 11% | 42% | 46% |

**Note**: values obtained by multiplying the intervention effect transition probabilities by the residual effects showed in Appendix I, Table B second row.

By multiplying the baseline physical activity distribution by G, we finally obtain the time=2 (two cycles after the intervention ended) physical activity distribution shown at point H:

1. Physical activity distribution of at **time 2** – high-income group1

| HIGH INCOME | **INA** | **INS** | **MOD** | **ACT** |
| --- | --- | --- | --- | --- |
|  | 6.19% | 25.17% | 43.03% | 25.61% |

# Appendix IV R code

# Baseline distribution of physical activity levels (table 1)

baseline_PA <- matrix(c(0.15, 0.2, 0.3, 0.35,

0.25, 0.3, 0.25, 0.2), byrow=T, ncol=4)

rownames(baseline_PA) <- c("High Income", "Low income")

colnames(baseline_PA) <- c("INA","INS","MOD","ACT")

baseline_PA

# Natural transition probabilities in absence of intervention (identity matrix)

Pnat <- diag(4)

rownames(Pnat) <- c("INA","INS","MOD","ACT")

colnames(Pnat) <- c("INA","INS","MOD","ACT")

Pnat

# High income subgroup intervention effect matrix (Table 2)

HI_effect <- matrix(c(0.15,0.48,0.31,0.06,

0.08,0.39,0.42,0.11,

0.03,0.22,0.51,0.24,

0.02,0.11,0.44,0.43), byrow=T, ncol=4)

rownames(HI_effect) <- c("INA","INS","MOD","ACT")

colnames(HI_effect) <- c("INA","INS","MOD","ACT")

HI_effect

# Low income subgroup intervention effect matrix (Table 3)

LI_effect <- matrix(c(0.45,0.38,0.16,0.01,

0.09,0.40,0.41,0.10,

0.02,0.14,0.47,0.37,

0.03,0.20,0.50,0.27),byrow=T, ncol=4)

rownames(LI_effect) <- c("INA","INS","MOD","ACT")

colnames(LI_effect) <- c("INA","INS","MOD","ACT")

LI_effect

# post-intervention distribution of physical activity levels

post_int_PA <- baseline_PA

post_int_PA[1,] <- baseline_PA[1,] %*% Pnat %*% HI_effect

post_int_PA[2,] <- baseline_PA[2,] %*% Pnat %*% LI_effect

post_int_PA

# double check whether all rowsums are equal to 1:

rowSums(baseline_PA)

rowSums(HI_effect)

rowSums(LI_effect)

rowSums(post_int_PA)

######################################################################################

# regression coefficients:

weibull_res <- data.frame(variable=c("lngamma",

"cons","inaLOW",

"insHIGH","insLOW",

"modHIGH","modLOW",

"actHIGH","actLOW"),

coefficient=c(0.780,

-4.093,0.055,

-0.125,-0.101,

-0.255,-0.225,

-0.522,-0.502),

se=c(0.008,

0.049,0.048,

0.028,0.447,

0.031,0.052,

0.035,0.064))

# survival parameters:

weibull_res$surv_par <- ifelse(weibull_res$variable %in% c("lngamma","cons"),

exp(weibull_res$coefficient),

exp(weibull_res$coefficient + weibull_res$coefficient[2]))

gamma <- weibull_res[1,4]

###########################################################################

# decay of effect probabilities:

decay_prob <- matrix(nrow=3, ncol=9)

decay_prob[,1] <- 1:3

for (j in 2:9) {

for(i in 1:3) {

decay_prob[i,j] <- 1-exp(weibull_res[j,4]*((i-1)^gamma -

i^gamma))

}

}

rownames(decay_prob) <- c("t+1","t+2","t+3")

colnames(decay_prob) <- c("cycle","inaHIGH","inaLOW","insHIGH","insLOW",

"modHIGH","modLOW","actHIGH","actLOW")

decay_prob

# residual effect probabilities:

resid_prob <- decay_prob

(resid_prob[,2:9] <- 1-decay_prob[,2:9])

##################################################################

# distribution of physical acitivity levels

post_int_PA

# inverse of matrices

(HI_post_int_PA <- solve(HI_effect))

(LI_post_int_PA <- solve(LI_effect))

HI_post_int_PA1 <- HI_post_int_PA

for(i in 1:4) {

HI_post_int_PA1[i,] <- HI_effect[i,]*resid_prob[1,(i*2)]

}

# make diagonal 1-remaining rowsum:

diag(HI_post_int_PA1) <- 1-(rowSums(HI_post_int_PA1)-diag(HI_post_int_PA1))

# post-intervention distribution (only for high-income now, but can follow these

# steps for low-income.

post_int_PA[1,] %*% HI_post_int_PA %*% HI_post_int_PA1

# can add cycles by adding another loop on top above or do it seperately,

# whatever you prefer. And good exercies to pracice some coding :)

######################################################################

### loop over cycles

num_cycles<-4

#### high income

theta_high<-vector("list", length=num_cycles+1)

### needs + 1 for length as first element of list is time 0, second is cycle 1, third is cycle 2 etc.

resid_high<-vector("list", length=num_cycles+1)

theta_high[[1]]<-post_int_PA[1,]

resid_high[[1]]<-HI_effect

for (j in 2:(num_cycles)){

resid_high[[j]]<-matrix(nrow=4, ncol=4)

for(i in 1:4){resid_high[[j]][i,]<- resid_high[[j-1]][i,]*resid_prob[j-1,(i*2)]}

diag(resid_high[[j]])<-1-(rowSums(resid_high[[j]])-diag(resid_high[[j]]))

theta_high[[j]]<-theta_high[[j-1]] %*% solve(resid_high[[j-1]]) %*% resid_high[[j]]

}

theta_high[[5]]<-theta_high[[4]] %*% solve(resid_high[[4]])

#### low income

theta_low<-vector("list", length=num_cycles+1)

resid_low<-vector("list", length=num_cycles+1)

theta_low[[1]]<-post_int_PA[2,]

resid_low[[1]]<-LI_effect

for (j in 2:(num_cycles)){

resid_low[[j]]<-matrix(nrow=4, ncol=4)

for(i in 1:4){resid_low[[j]][i,]<- resid_low[[j-1]][i,]*resid_prob[j-1,(i*2+1)]}

diag(resid_low[[j]])<-1-(rowSums(resid_low[[j]])-diag(resid_low[[j]]))

theta_low[[j]]<-theta_low[[j-1]] %*% solve(resid_low[[j-1]]) %*% resid_low[[j]]

}

theta_low[[5]]<-theta_low[[4]] %*% solve(resid_low[[4]])

theta_high

theta_low
